# Decreased PERP Expression on Peripheral Blood Mononuclear Cells from Patient with Rheumatoid Arthritis Negatively Correlates with Disease Activity

**DOI:** 10.1155/2013/256462

**Published:** 2013-08-26

**Authors:** Yanchun Du, Lin Deng, Yan Li, Lu Gan, Yantang Wang, Guixiu Shi

**Affiliations:** ^1^The First Hospital of Xiamen University, Xiamen 361003, China; ^2^Division of Rheumatology, State Key Laboratory of Biotherapy, West China Hospital, Sichuan University, Chengdu 610041, China; ^3^British Heart Foundation Glasgow Cardiovascular Research Centre, Institute of Cardiovascular and Medical Sciences, University of Glasgow, Glasgow G12 8TA, UK; ^4^Department of Immunology, Chengdu Medical College, Chengdu 610000, China; ^5^Department of Rheumatology and Clinical Immunology, The First Hospital of Xiamen University, Xiamen 361003, China

## Abstract

*Background*. PERP, p53 apoptosis effector related to PMP-22, is a p53-dependent apoptosis in diverse cell types and has cell type-specific roles in p53-mediated apoptosis. However, its role in PBMCs of RA patients has remained largely unclear. *Objectives*. The aim of this study was to detect the expression levels of PERP on PBMCs of RA patients and healthy controls and analyze the role of PERP in the pathogenesis of RA. *Methods*. The mRNA expression levels of PERP and IL-17 were detected by real-time PCR in PBMCs from patients with RA (*n* = 40) and healthy controls (*n* = 40). The correlations of PERP expression levels to IL-17 transcripts and disease activity parameters were analyzed. *Results*. The PERP and IL-17 expression levels in the PBMCs were significantly decreased and increased in comparison of which in healthy controls. The mRNA expression levels of PERP in PBMCs from patients with RA were negatively correlated with IL-17 and disease activity parameters DAS28, RF, CRP, and ESR rather than Anti-CCP and ANA. *Conclusions*. These results demonstrated that PERP might be involved in the pathogenesis and a potential therapeutic target of RA by regulating the expression of IL-17.

## 1. Introduction

Rheumatoid arthritis (RA) is a systemic autoimmune disease characterized by chronic joint inflammatory of synovial tissues in multiple joints and destruction of articular cartilage and bone [1]. The abnormalities of immune regulation and function are implicated in the pathogenesis of RA. Activation and recruitment of immune cells, especially lymphocytes and monocytes, into joints are characteristic features of this disease [2]. In normal inflammatory responses, proper apoptosis is critical for maintaining the lymphocyte homeostasis and tissue growth by initiation of apoptotic cascades [3]. Furthermore, the immune system heavily depends on apoptosis to ameliorate inflammation for preventing misdirected damage to normal tissues [4]. However, defection of the apoptotic cascades usually contributes to human autoimmune diseases, such as rheumatoid arthritis (RA) [5]. 

PERP, as a tetrapan protein, was first identified as a p53 transcriptional target proapoptotic gene which expresses high levels during apoptosis rather than cell cycle arrest and has cell-specific and tissue-specific roles in p53-mediated apoptosis [6, 7]. PERP is a member of peripheral myelin protein 22/growth arrest specific 3 (PMP-22/gas3) family, which includes PMP-22 and the epithelial membrane proteins 1, 2, and 3 [8]. The apoptotic mechanism of PERP may differentiate with the BH3-containing proteins such as Noxa and Puma, since it localizes to the plasma membrane and secretory pathway rather than the mitochondria [9]. Prior study demonstrates that PERP contributes to radiation-induced apoptosis in CD4^+^CD8^+^ thymocytes which undergo a well-characterized p53-dependent apoptotic response using *PERP* ^−/−^ mice [6, 10]. In autoimmune diseases, researcher found that the tumor suppressor molecule p53, which is significant in apoptosis and cell-cycle control, is greatly reduced in peripheral blood mononuclear cells from patients with RA, systemic lupus, and multiple sclerosis compared with that in normal controls [11]. This observation demonstrates that the decreased expression of p53 might contribute to the pathogenesis of autoimmune diseases by failing to eliminate potentially pathogenic cells. However, the role of PERP in autoimmune diseases is still unknown.

Interleukin-17 (IL-17) is a proinflammatory cytokine predominantly produced by a specific subset of CD T helper cells called Th17 cells and is critical for inducing and perpetuating chronic inflammation, cartilage damage, and bone erosion [12, 13]. Several studies have suggested that IL-17 plays a key role in the pathogenesis of RA [14, 15], and the expression of IL-17 gene is associated with the inflammatory process and disease activity of RA disease [16]. Recently, two investigational monoclonal antibodies which neutralize IL-17 appeared to be safe and effective in the early stage clinical trials in RA patients [17, 18]. The expression of IL-17 can downregulate apoptosis in RA and p53 can regulate Th1 and Th17 functions in patients with RA participating in the pathogenesis of RA [19, 20]. However, as a p53 transcriptional target, the role of PERP in regulating IL-17 and involving in the pathogenesis of RA is still unclear.

The aim of this study was to investigate the role of PERP in disease activity and progression of human and to explore the possibility of PERP p as a target for the treatment of RA. We measured the mRNA expression levels of PERP and IL-17 in the peripheral blood mononuclear cells (PBMCs) from RA patients and analyzed the correlation between them. Furthermore, we investigated the correlation of PERP expression levels with different activity parameters. The results showed that the expression of PERP mRNA in the peripheral blood mononuclear cells (PBMCs) from patients with RA was significantly decreased compared with healthy controls (HCs). The expression levels of PERP were negatively correlated with IL-17 mRNA levels in PBMCs and the disease activity parameters including disease activity scores (DAS28), rheumatoid factor (RF), C-reaction antibodies (anti-CCP), and erythrocyte sedimentation rate (ESR). These data suggested that the reduced expression of PERP might participate in regulating IL-17 and involving in the pathogenesis of RA.

## 2. Materials and Methods

### 2.1. Patients and Controls

A total of 40 patients with rheumatoid arthritis (RA) (5 men and 35 women, age 23–76, mean 45.3 years), all from the outpatient clinic of the Department of Rheumatology, West China Hospital, Sichuan University, were included in the present study. The RA patients were diagnosed based on the 1987 criteria of the American College of Rheumatology [21]. The disease duration of RA patients was between 2 months and 12 years. Some patients were taking nonsteroidal anti-inflammatory drugs. None of the patients had ever taken disease-modifying antirheumatic drugs. Patients taking corticosteroids or vitamin D and those who had renal insufficiency were excluded. The study was approved by the Ethics Committee of West China Hospital. All procedures involving specimens obtained from human were performed with informed consent from each patient. The disease activity score calculated for 28 joints (DAS28) [22], has been widely used in clinical trials and for the assessment of patients in the clinic to monitor disease activity of patients with RA. Healthy controls peripheral blood mononuclear cells (PBMCs) were obtained from 40 healthy volunteers (5 men and 35 women, age 28–63, mean 43.8 years). The demographic and clinical characteristics of healthy controls and patients with RA were summarized in Table 1. 

### 2.2. PBMCs Isolation

Peripheral blood mononuclear cells (PBMCs) were purified from peripheral blood of healthy controls and patients by standard density-gradient centrifugation, using Ficoll-Paque Plus (Axis-Shied PoC AS, Oslo, Norway). Briefly, blood was centrifuged at 500 g for 20 min at room temperature. After centrifugation, the top plasma layer was collected and stored at −80°C. Then the precipitation was diluted 1 : 1 with sterile phosphate buffered saline (PBS) before to layering over the Ficoll. PBMCs were carefully aspirated with spinning at 1600 rpm for 30 min. The cells were then washed three times with PBS before adding trizol (Invitrogen, USA). 

### 2.3. RNA Extraction and Real-Time PCR

Total RNA extracted from PBMCs was extracted with Trizol (Invitrogen, USA) following the manufacturer's instructions. Briefly, 1 *μ*g total RNA was first reverse-transcribed to cDNA with reverse transcription reagent kits according to the manufacturer's protocol (Bio-Rad, Hercules, CA, USA). A 20 *μ*L reverse-transcribed reaction mixture was acted including 4 *μ*L 5×iScript reaction mix, 1 *μ*L iScript reverse transcriptase and 15 *μ*L with nuclease-free water, and 1 *μ*g total RNA. The reaction conditions were: 25°C for 5 min, then 42°C 30 mins, and last 85°C 5 min. The expression levels of *PERP, IL-17A,* and *GAPDH *were determined by real-time quantitative PCR, using SsoFast EvaGreen Supermix (Bio-Rad, Hercules, CA, USA). A 10 *μ*L SsoFast EvaGreen PCR reaction mixture was used containing 2 *μ*L of cDNA, 0.2 *μ*L of sense primer, 0.2 *μ*L of antisense primer, 2.6 *μ*L ddH_2_O, and 5 *μ*L SsoFast EvaGreen Supermix. Quantitative PCR was performed on the iQ5 and MyiQ Real-Time PCR Detection Systems (Bio-Rad, Hercules, CA, USA). The PCR reaction conditions were: 95°C for 1 min for denature, followed by 40 cycles of three-step PCR including melting for 10 s at 95°C, annealing for 10 s at 60°C, and extending for 10 s at 72°C. The mRNA expression was normalized to the expression levels of *GAPDH*, and relative expression was calculated with the 2^−ΔΔCt^ method. The following sense and antisense primers were used: for PERP, sense 5′-AGAGCCTCATGGAGTACGC-3′ and 5′-CCTCACTTGCCGAAACAGC-3′; for IL-17A, sense 5′-CTACAACCGATCCACCTCAC-3′ and 5′-TGTGGTAGTCCACGTTCCCAT-3′; for GAPDH, sense 5′-AGAAGGCTGGGGCTCATTTG-3′ and antisense 5′-AGGGGCCATCCACAGTCTTC-3′.

### 2.4. Statistical Analysis

The statistical significance of the data was analyzed by PRISM software (GraphPad Software, San Diego, CA, USA) using Mann-Whitney test. Spearmen's rank test was utilized to test the correlations between the levels of PERP expression and clinical parameters of RA patients. *P* values <0.05 were considered significant. 

## 3. Results

### 3.1. Decreased Expression of PERP in PBMCs from Patients with RA

**Figure 1:**
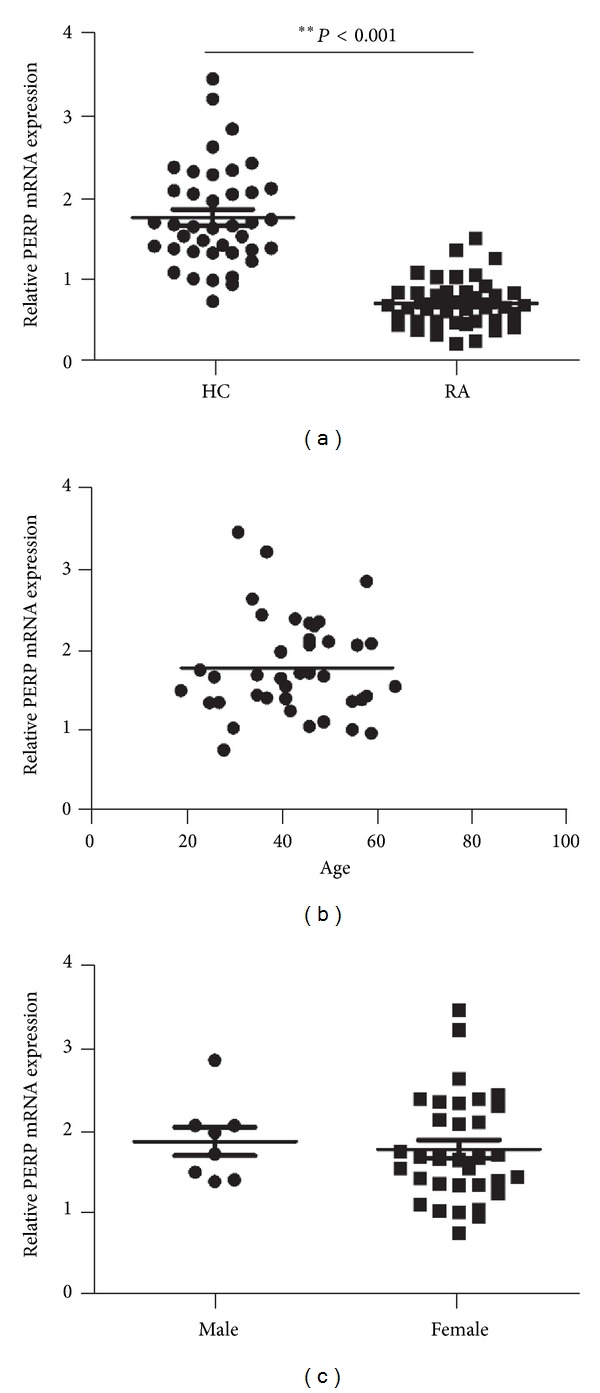
Decreased expression of PERP in peripheral blood lymphocytes (PBMCs) from patients with rheumatoid arthritis (RA) and PERP expression levels in age and sex in healthy controls (HCs) have no differences. (a) Expression of PERP mRNA in PBMCs from RA patients (*n* = 40) and healthy controls (HCs; *n* = 40) (*P* < 0.05). (b) The correlation between age and PERP expression levels in healthy control (HCs; *n* = 40) (*P* > 0.05). (c) The expression levels of Perp between male and female in healthy control (HCs; *n* = 40) (*P* > 0.05). *P* value was determined by Mann-Whitney test. *P* < 0.05 deems significantly different.

Peripheral blood mononuclear cells (PBMCs) were separated from 40 RA patients and 40 age and sex matched healthy controls. Quantitative-Real-time PCR was used to analyze the mRNA expression level of PERP. Results showed that Perp transcripts were significantly lower in RA patients than healthy controls (Figure 1(a)) (*P* < 0.001), while the expression of Perp mRNA levels in healthy controls (HCs) have no correlations with age and sex (Figures 1(b) and 1(c)) (*P* > 0.05). 

### 3.2. Expression Levels of PERP in PBMCs Correlated with Parameters of Disease Activity in Patients with RA

**Figure 2:**
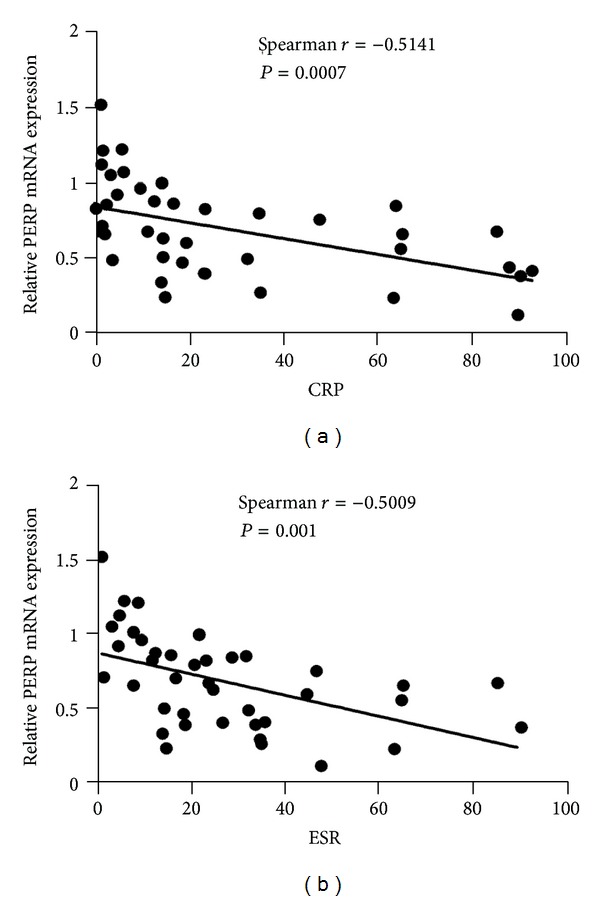
Expression of PERP on PBMCs from RA patients correlates with CRP and ESR. The expression levels of PERP in PBMCs of patients with RA negatively correlate with C-reactive protein (CRP) and erythrocyte sedimentation rate (ESR) (*P* < 0.01; *P* < 0.01). *P* < 0.05 deems significantly different.

**Figure 3:**
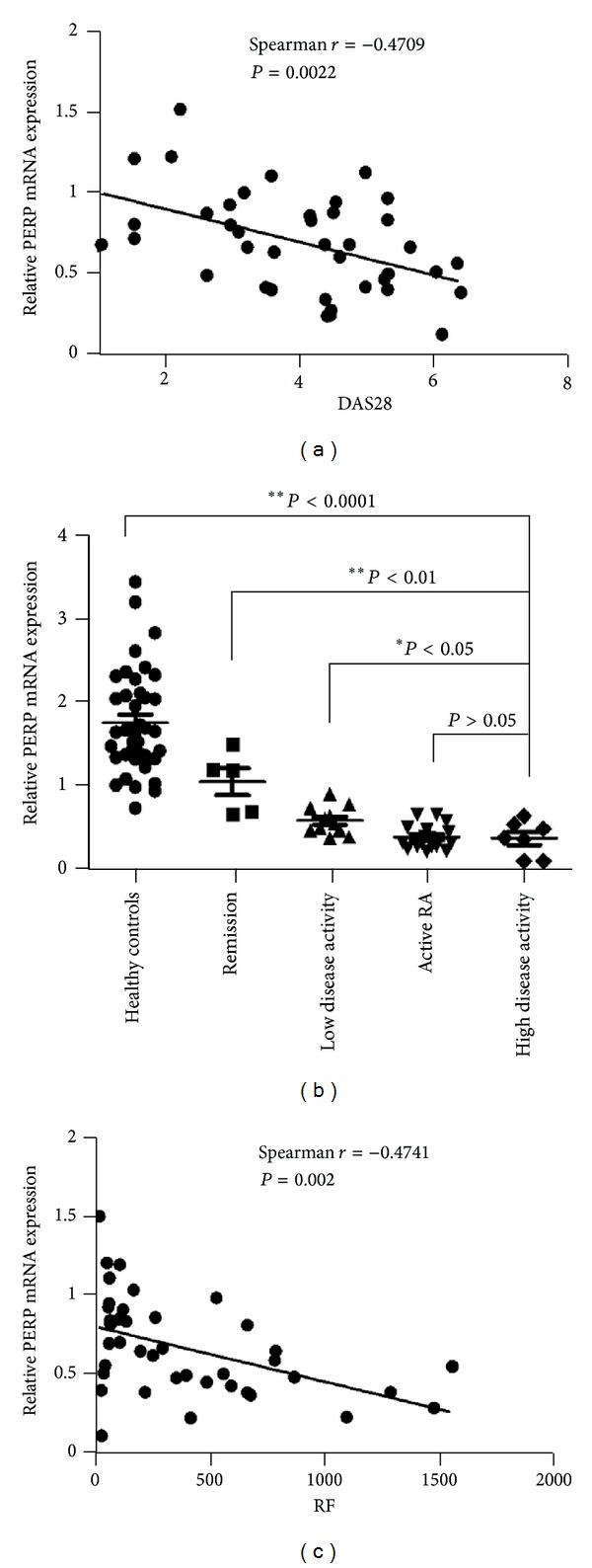
Correlations of PERP mRNA expression on PBMCs in RA patients with DAS28 and RF. PERP mRNA transcripts negatively correlate with disease activity score (DAS28) (a) (*P* < 0.01) and rheumatoid factor (RF) (c) (*P* < 0.01). (b) The expression levels of PERP of PBMCs from healthy controls (HCs; *n* = 40) and RA patients in different stages of disease activity determined by DAS28. *P* < 0.05 deems significantly different.

**Figure 4:**
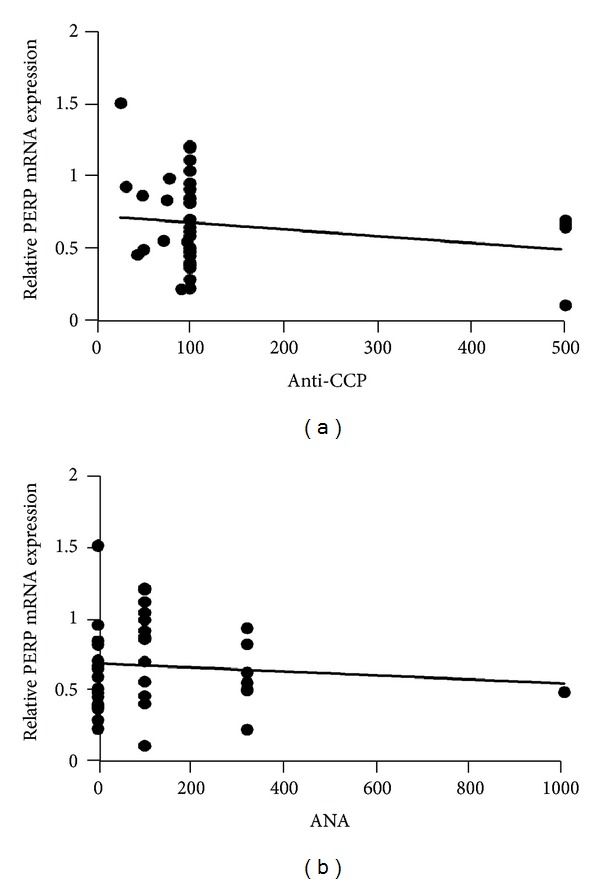
Correlations of PERP mRNA expression on PBMCs in RA patients with DAS28 and RF. The PERP mRNA expression levels do not have correlations with anti-cyclic citrullinated protein antibodies (anti-CCP) (a) (*P* > 0.05) and antinuclear antibodies (ANA) (b) (*P* > 0.05). *P* < 0.05 deems significantly different.

In order to detect whether the low expression levels of PERP may involve in the disease pathogenesis and progression of RA, we analyzed the correlation between PERP transcripts and different disease activity parameters. A significant negative correlation was seen between PERP mRNA levels in RA patients and serologic parameters of disease activity, including CRP (Figure 2(a)) (*P* = 0.0066), ESR (Figure 2(b)) (*P* = 0.0014), DAS28 (Figure 3(a)) (*P* = 0.0014), and RF (Figure 3(c)) (*P* = 0.0008). However, the PERP mRNA expression levels have no correlations with anti-CCP (Figure 4(a)) (*P* > 0.05) and ANA (Figure 4(b)) (*P* > 0.05). There were no correlations between the expression levels of PERP in RA patients and sex, age, or duration of RA. (data not shown) (*P* > 0.05). We define the disease activity states criteria of RA by disease activity score in 28 joints (DAS28), with remission (DSA28 < 2.4), low disease activity (DAS28 between 2.4 and 3.6), active RA (DAS28 between 3.6 and 5.5), and high disease activity (DAS28 > 5.5) [23]. Then we compared the PERP expression in PBMCs from RA patients and healthy controls. It is showed that the high disease activity group had the lowest expression of PERP compared with remission group, low disease activity group, and healthy controls group. However, there was no statistical significance between active RA group and high disease activity group (Figure 3(b)). 

### 3.3. The Correlation between the Expression of PERP and IL-17 in RA Patients

**Figure 5:**
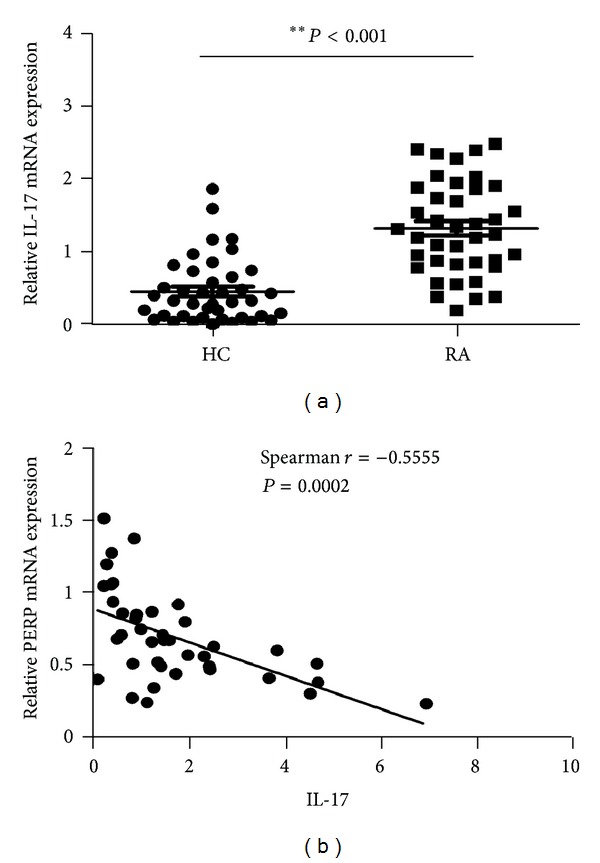
Correlations of PERP and IL-17 mRNA expression levels in RA patients. The mRNA expression levels of patients with RA (*n* = 40) were determined by real-time PCR. (a) The increased expression levels of PERP in patients with RA (*P* < 0.01). (b) There is a significant negative correlation between PERP and IL-17 mRNA expression levels (*P* < 0.01). *P* < 0.05 deems significantly different.

The prior study demonstrates that Th17 cells and their specific transcription factor or related cytokines are being recognized as important mediators in inflammatory and autoimmune diseases including RA [24]. To assess the relationships between the mRNA levels of PERP and IL-17 in RA patients, we first detected the IL-17 mRNA expression levels on PBMCs in healthy control, (HCs) and patients with RA, and then we examined the correlation between the mRNA levels of PERP and IL-17 in PBMCs of RA patients. The IL-17 mRNA expression levels were significantly increased (Figure 5(a)) (*P* < 0.01), and there was a significantly negative correlation between the expression of PERP and IL-17 (Figure 5(b)) (*P* < 0.01).

## 4. Discussion 

Apoptosis is an evolutionarily conserved, multi-step processes cell death pathway which occurs in a variety of physiological conditions. The balance of apoptosis is main mechanism of physiological cell death, which is significant for homeostasis in multi-cellular organisms including the immune system [4]. Abnormal elevates in apoptosis can result to immunodeficiency and a failure to undergo apoptosis can contribute to the development of autoimmunity [25, 26]. In RA, apoptosis plays divergent roles in the pathogenesis of this disease. In joint of acute patients, there are fewer apoptotic cells than healthy controls, and experimental data demonstrate that elevated apoptosis in the joint contributes to beneficial roles [27]. The impaired of PBMCs apoptosis also detected in RA patients [28]. 

Previous studies have shown that the expression of p53 was decreased in PBMCs from patients with RA [11] and there are p53 mutations in rheumatoid arthritis synovium [29]. The defects in downstream p53 target gene play a vital role in promoting systemic autoimmunity diseases. For example, p21, a downstream cyclin dependent kinase inhibitor and transcriptional target of p53, is also downexpressed in patients with RA [30]. PERP, another direct p53 target gene, may also play an important role in human RA disease. In this study, we first examined the PERP expression levels on PBMCs in patients with RA compared with healthy controls and further explored their correlation with disease activity parameters. Results showed that PERP mRNA expression levels in PBMCs from patients with RA were significantly decreased compared with healthy controls. Furthermore, we found that the PERP mRNA expression levels were inversely correlated with disease activity referred to as disease activity score 28 (DAS28), C-reactive protein (CRP), erythrocyte sedimentation rate (ESR), and rheumatoid factor (RF). Although there are no correlation relationships with anti-CCP and ANA, the prior studies demonstrated that anti-CCP and ANA have no significant relationship with both disease activity and severity [31, 32].

Proper apoptosis of lymphocytes was vital for tolerance and autoimmunity, and insufficient apoptosis of lymphocytes contributes to the persistence of RA disease [5]. It seems plausible that PERP mRNA expression levels reflect the disease severity since the negative correlation with disease activity parameters of RA. To further explore whether PERP expression can serve as a biomarker for disease activity in RA, we choose DAS28 as criteria to define the disease activity states and analyze the relations with PERP mRNA expression levels and each disease activity state. The results showed that the high disease activity group has statistical significance with remission group and low disease activity group. However, there is no difference between active RA group and high disease activity group. The data above suggest that PERP might be a potential diagnostic marker for RA severity and as a therapeutic target for treatment. 

Interleukin-17 (IL-17) was first discovered in 1993 and found exerting various biological functions in vivo that might be involved in the pathogenesis of a wide range of inflammatory, infectious and autoimmune diseases [13, 33]. Previous study showed that IL-17 expression has associated with disease activity and make IL-17 as a key player in RA pathogenesis [34, 35]. Latest study demonstrated IL-17 gene expression in PBMCs of patients with RA is significantly higher than healthy controls and suggested that IL-17 had an important role in the pathogenesis of RA [16]. Also the tumor suppressor gene p53 can regulate Th17 functions by inhibiting production of IL-17 in patients with RA and participate in the pathogenesis of RA [20], and the downstream of p53 transcriptional target gene *Bax* involved in the effect of sulforaphane (SFN) inhibit the production of IL-17 by rheumatoid T cells in vitro [36]. In order to explore the potential mechanism of PERP, which is another transcriptional target beside *Bax*, participate in the pathogenesis of RA, and then we detect the mRNA expression levels of IL-17 on PBMCs of patients with RA and analyzed the expression correlation between them. The result showed that the IL-17 transcripts increased significantly in patients with RA compared with healthy controls, and have significantly negative correlation with the Perp mRNA expression levels. This data demonstrate that Perp may be one of the regulators of IL-17 expression participating in the pathogenesis of RA. 

Collectively, the expression of PERP is downregulated on peripheral blood mononuclear cells (PBMCs) from patients with RA, which significantly inverse-correlated with IL-17 gene expression levels in PBMCs and disease activity. Our data suggest that PERP might be a regulator of IL-17 participating in the pathogenesis of RA and a potential diagnostic marker for RA severity and a therapeutic target for treatment. However, the exact role of PERP in RA disease still needs to be further elucidated in the future.

## Figures and Tables

**Table 1:** Demographic and clinical characteristics of the patients with rheumatoid arthritis (RA) and healthy control (HC) subjects.

	RA patients (*n* = 40)	HCs (*n* = 40)
Age, mean (range) years	45.3 (23–76)	43.8 (28–63)
Male : female	5 : 35	8 : 32
Rheumatoid factor (RF) (IU/mL)	362.1 (22.6–1560)	21.34 (19.3–43.8)
DAS28 mean (range) score	4.05 (1.05–6.59)	—
C-reactive protein (CRP) (mg/L)	27.1 (0.3–90.6)	—
Erythrocyte sedimentation rate (ESR)	26.9 (1.25–90.9)	—
Disease duration, mean (range) months	28.4 (2–136)	—

DAS28: disease activity score 28.
